# 1-(2-Methyl-5-nitro-1*H*-imidazol-1-yl)propan-2-yl acetate

**DOI:** 10.1107/S1600536814002505

**Published:** 2014-02-15

**Authors:** Hafiz Abdullah Shahid, Ejaz Hussain, Sajid Jahangir, Sammer Yousuf

**Affiliations:** a Department of Chemistry, Faculty of Science, Federal Urdu University of Arts, Science and Technology Gulshan-e-Iqbal, Karachi 75300, Pakistan; b H.E.J. Research Institute of Chemistry, International Center for Chemical and Biological Sciences, University of Karachi, Karachi 75270, Pakistan

## Abstract

In the title compound, C_9_H_13_N_3_O_4_, an ester of the anti-infection drug secnidazole, the dihedral angle between the nitro­imidazole mean plane (r.m.s. deviation = 0.028 Å) and the pendant acetate group is 43.17 (11)°. In the crystal, inversion dimers linked by pairs of C—H⋯O inter­actions generate *R*_2_^2^(10) loops and further C—H⋯O hydrogen bonds link the dimers into [100] chains. Weak aromatic π–π stacking inter­actions with a centroid–centroid distance of 3.7623 (11) Å are also observed.

## Related literature   

For background to the anti­bacterial properties of nitro­imidazole and secnidazole-like compounds, see: Mital (2009 ▶); Edwards (1993 ▶); Crozet *et al.* (2009 ▶). For the crystal structures of related compounds, see: Yousuf *et al.* (2013 ▶); Tao *et al.* (2008 ▶);Zeb *et al.* (2012 ▶). 
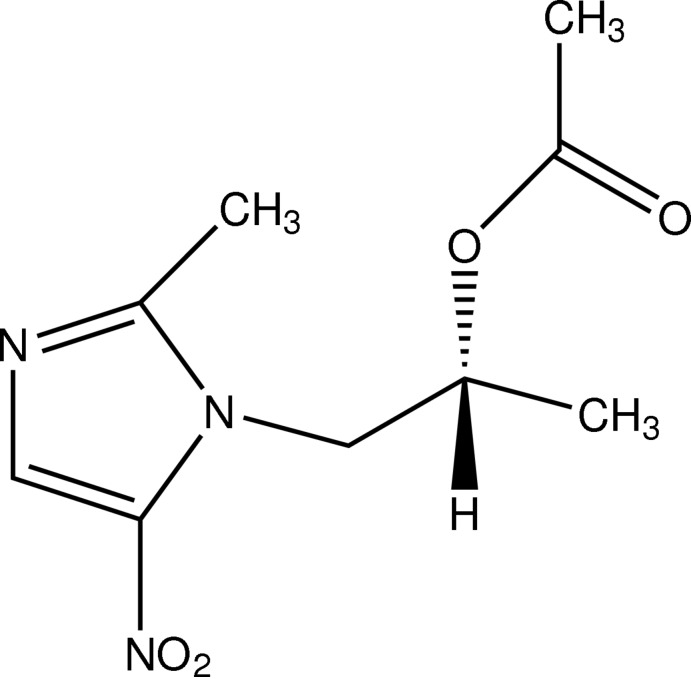


## Experimental   

### 

#### Crystal data  

C_9_H_13_N_3_O_4_
*M* *_r_* = 227.22
Monoclinic, 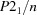

*a* = 6.1771 (5) Å
*b* = 8.9928 (7) Å
*c* = 20.3736 (16) Å
β = 90.978 (2)°
*V* = 1131.58 (16) Å^3^
*Z* = 4
Mo *K*α radiation
μ = 0.11 mm^−1^
*T* = 273 K
0.45 × 0.27 × 0.06 mm

#### Data collection  

Bruker SMART APEX CCD diffractometer
Absorption correction: multi-scan (*SADABS*; Bruker, 2000 ▶) *T*_min_ = 0.954, *T*_max_ = 0.994
6541 measured reflections
2042 independent reflections
1567 reflections with *I* > 2σ(*I*)
*R*_int_ = 0.025

#### Refinement  

*R*[*F* ^2^ > 2σ(*F* ^2^)] = 0.043
*wR*(*F* ^2^) = 0.119
*S* = 1.02
2042 reflections
145 parameters
H-atom parameters constrained
Δρ_max_ = 0.20 e Å^−3^
Δρ_min_ = −0.12 e Å^−3^

### 

Data collection: *SMART* (Bruker, 2000 ▶); cell refinement: *SAINT* (Bruker, 2000 ▶); data reduction: *SAINT*; program(s) used to solve structure: *SHELXS97* (Sheldrick, 2008 ▶); program(s) used to refine structure: *SHELXL97* (Sheldrick, 2008 ▶); molecular graphics: *SHELXTL* (Sheldrick, 2008 ▶); software used to prepare material for publication: *SHELXTL*, *PARST* (Nardelli, 1995 ▶) and *PLATON* (Spek, 2009 ▶).

## Supplementary Material

Crystal structure: contains datablock(s) global, I. DOI: 10.1107/S1600536814002505/hb7195sup1.cif

Structure factors: contains datablock(s) I. DOI: 10.1107/S1600536814002505/hb7195Isup2.hkl

Supporting information file. DOI: 10.1107/S1600536814002505/hb7195Isup3.cml

CCDC reference:

Additional supporting information:  crystallographic information; 3D view; checkCIF report

## Figures and Tables

**Table 1:** Hydrogen-bond geometry (Å, °)

*D*—H⋯*A*	*D*—H	H⋯*A*	*D*⋯*A*	*D*—H⋯*A*
C4—H4*A*⋯O1^i^	0.93	2.45	3.369 (2)	168
C6—H6*A*⋯O4^ii^	0.97	2.53	3.460 (2)	161

Symmetry codes: (i) 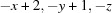; (ii) 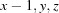.

**Fig. 1.**
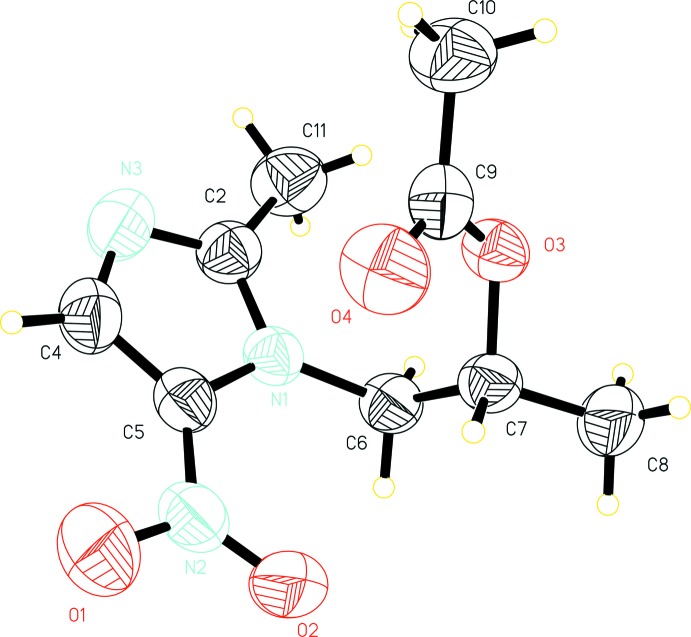
Fig:1 The molecular structure of title compound I, showing displacement ellipsoids drawn at 50% probability level.

**Fig. 2.**
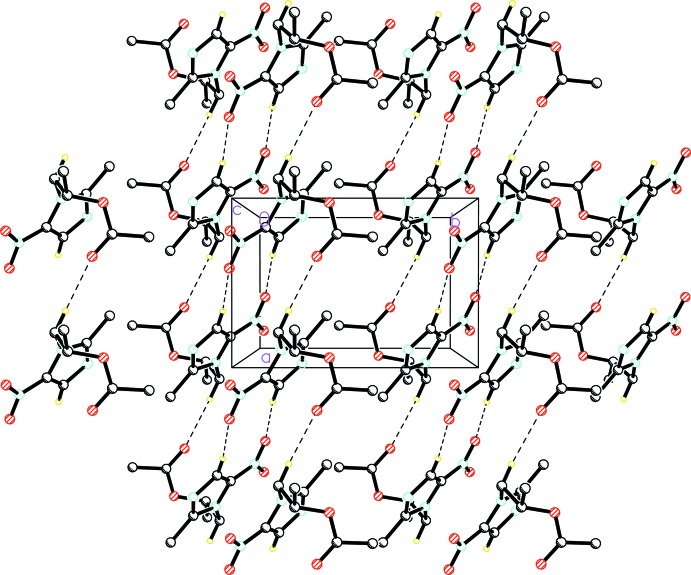
Fig: 2 Crystal packing diagram, showing intermolecular hydrogen bonding as dashed lines.

## supplementary crystallographic information

### 1. Comment

The title compound (I) is an ester derivative of well known 5-nitroimidazole drug i.e secnidazole. The worthwhile use of nitroimidazole derivatives is in the treatment of diseases caused by protozoa and anaerobic bacteria (Mital, 2009). Members of nitroimidazole drugs are pronounced in thier wide-range activities and in addition during their use the rate of resistance in anaerobes is still very low (Edwards, 1993). Antiprotozoal and bactericidal properties of nitroimidazoles are associated with their aromatic nitro group. The Secnidazole like chemotherapeutic agents inhibit the growth of both anaerobic bacteria and some anaerobic protozoa (Crozet *et al.* 2009).

The structure of the title compound (I) is similar to our previously reported compound 1-(2-Methyl-5-nitro-1*H*-imidazol-1-yl)acetone with the difference that acetone moiety is replaced by propyl acetate group (C6—C10/O3,O4)(Yousuf *et al.* 2013);. It also exhibits bond lengths and angles that are of normal range (Yousuf *et al.* 2013); A three dimensional consolidated architecture is formed by the non-covalent interactions of molecules in the crystal *via* C4– H4A···O1 [2.45 Å], and C6– H6A···O4 [2.53 Å] hydrogen bonding with *R*_2_^2^(10) ring motifs. Possible weak pi-pi interactions (*Cg*1···*Cg*1) with minimum centroid-centroid distance of 3.7623 (11) Å are also observed.

### 2. Experimental

The title compound was synthesized by adding acetic anhydride (1.2 ml, 12.70 mmol)to a hot (70 °C) stired solution of secnidazole (2 g m, 10.8 mmol) in pyridine (2 ml) and toluene (10 ml). The reaction mixture was further processed to refluxed for 5 hrs, cooled, treated with water and then organic phase was evaporated to obtain solid product which was recrystallized from chloroform and toluene solution to yield greenish plates in 81% yield. Melting point 346–348 K. ^1^H NMR (300 MHz, DMSO-d6): δ 8.006 (s, 1 H, imidazole H), 5.162–5.089 (m, 1 H, CH), 4.573–4.322 (m, 2 H, CH_2_), 3.300 (s, 3 H, CH_3_), 1.856 (s, 3 H CH_3_), 1.265–1.244 (d, J=6.3 Hz, 3 H, CH_3_). ^13^C NMR (75 MHz, DMSO-d6): δ 169.35 (C=O), 151.52 (N=C), 138.40 (C—NO_2_), 133.01 (N—CH), 68.64 (O—CH), 49.31 (N—CH_2_), 20.44 (CH_3_), 17.11 (CH_3_), 13.93 (CH_3_). IR (neat, cm^-1^): 3434, 3122, 2994, 1732, 1532, 1368, 1140, 1080.

### 3. Refinement

The hydrogen atoms are positioned at their calculated positions geometrically with C—H = 0.9300 Å, 0.9600 Å, 0.9700 Å, 0.9800 Å for aromatic, methyl, methylen, and methin H respectively. These are constrained to ride on their parent atoms during subsequent refinement with *U*_iso_(H) = 1.2*U*_eq_(C) for methyl, and *U*_iso_(H) = 1.5_eq_(C) for rest of the H atoms.

### Crystal data

**Table:**

C_9_H_13_N_3_O_4_	*F*(000) = 480
*M**_r_* = 227.22	*D*_x_ = 1.334 Mg m^−^^3^
Monoclinic, *P*2_1_/*n*	Mo *K*α radiation, λ = 0.71073 Å
*a* = 6.1771 (5) Å	Cell parameters from 1751 reflections
*b* = 8.9928 (7) Å	θ = 2.5–22.3°
*c* = 20.3736 (16) Å	µ = 0.11 mm^−^^1^
β = 90.978 (2)°	*T* = 273 K
*V* = 1131.58 (16) Å^3^	Plate, colourless
*Z* = 4	0.45 × 0.27 × 0.06 mm

### Data collection

**Table:**

Bruker SMART APEX CCD diffractometer	2042 independent reflections
Radiation source: fine-focus sealed tube	1567 reflections with *I* > 2σ(*I*)
Graphite monochromator	*R*_int_ = 0.025
ω scan	θ_max_ = 25.5°, θ_min_ = 2.0°
Absorption correction: multi-scan (*SADABS*; Bruker, 2000)	*h* = −7→7
*T*_min_ = 0.954, *T*_max_ = 0.994	*k* = −10→10
6541 measured reflections	*l* = −23→24

### Refinement

**Table:**

Refinement on *F*^2^	Primary atom site location: structure-invariant direct methods
Least-squares matrix: full	Secondary atom site location: difference Fourier map
*R*[*F*^2^ > 2σ(*F*^2^)] = 0.043	Hydrogen site location: inferred from neighbouring sites
*wR*(*F*^2^) = 0.119	H-atom parameters constrained
*S* = 1.02	*w* = 1/[σ^2^(*F*_o_^2^) + (0.0605*P*)^2^ + 0.1628*P*] where *P* = (*F*_o_^2^ + 2*F*_c_^2^)/3
2042 reflections	(Δ/σ)_max_ < 0.001
145 parameters	Δρ_max_ = 0.20 e Å^−^^3^
0 restraints	Δρ_min_ = −0.12 e Å^−^^3^

### Special details

**Table:**

Geometry. All e.s.d.'s (except the e.s.d. in the dihedral angle between two l.s. planes) are estimated using the full covariance matrix. The cell e.s.d.'s are taken into account individually in the estimation of e.s.d.'s in distances, angles and torsion angles; correlations between e.s.d.'s in cell parameters are only used when they are defined by crystal symmetry. An approximate (isotropic) treatment of cell e.s.d.'s is used for estimating e.s.d.'s involving l.s. planes.
Refinement. Refinement of *F*^2^ against ALL reflections. The weighted *R*-factor *wR* and goodness of fit *S* are based on *F*^2^, conventional *R*-factors *R* are based on *F*, with *F* set to zero for negative *F*^2^. The threshold expression of *F*^2^ > σ(*F*^2^) is used only for calculating *R*-factors(gt) *etc*. and is not relevant to the choice of reflections for refinement. *R*-factors based on *F*^2^ are statistically about twice as large as those based on *F*, and *R*- factors based on ALL data will be even larger.

### Fractional atomic coordinates and isotropic or equivalent isotropic displacement parameters (Å^2^)

**Table:**

	*x*	*y*	*z*	*U*_iso_*/*U*_eq_	
O1	0.9023 (2)	0.57210 (19)	0.08857 (8)	0.0881 (5)	
O2	0.6626 (3)	0.55701 (17)	0.16347 (8)	0.0834 (5)	
O3	0.48247 (17)	0.11443 (13)	0.18968 (6)	0.0498 (3)	
O4	0.8273 (2)	0.17014 (17)	0.16857 (8)	0.0744 (5)	
N2	0.7361 (3)	0.51890 (17)	0.11093 (9)	0.0604 (5)	
N3	0.5484 (3)	0.2475 (2)	−0.00606 (8)	0.0658 (5)	
N1	0.4442 (2)	0.33433 (15)	0.09081 (7)	0.0476 (4)	
C5	0.6300 (3)	0.40876 (19)	0.07274 (9)	0.0497 (5)	
C4	0.6890 (3)	0.3540 (2)	0.01383 (10)	0.0599 (5)	
H4A	0.8088	0.3851	−0.0095	0.072*	
C2	0.4036 (3)	0.2373 (2)	0.04109 (10)	0.0552 (5)	
C11	0.2199 (3)	0.1309 (3)	0.03950 (12)	0.0744 (6)	
H11A	0.2237	0.0738	−0.0003	0.112*	
H11B	0.0859	0.1848	0.0412	0.112*	
H11C	0.2312	0.0654	0.0766	0.112*	
C6	0.3239 (3)	0.3431 (2)	0.15215 (9)	0.0517 (5)	
H6A	0.1826	0.2979	0.1455	0.062*	
H6B	0.3018	0.4468	0.1633	0.062*	
C7	0.4387 (3)	0.26641 (19)	0.20886 (9)	0.0489 (5)	
H7A	0.5748	0.3178	0.2193	0.059*	
C8	0.2980 (4)	0.2624 (2)	0.26847 (10)	0.0674 (6)	
H8A	0.3741	0.2134	0.3038	0.101*	
H8B	0.1670	0.2092	0.2583	0.101*	
H8C	0.2633	0.3622	0.2814	0.101*	
C9	0.6837 (3)	0.0809 (2)	0.17030 (9)	0.0514 (5)	
C10	0.7003 (3)	−0.0780 (2)	0.15128 (12)	0.0731 (6)	
H10A	0.8452	−0.0989	0.1377	0.110*	
H10B	0.6007	−0.0982	0.1157	0.110*	
H10C	0.6658	−0.1395	0.1882	0.110*	

### Atomic displacement parameters (Å^2^)

**Table:**

	*U*^11^	*U*^22^	*U*^33^	*U*^12^	*U*^13^	*U*^23^
O1	0.0725 (10)	0.0951 (12)	0.0972 (12)	−0.0330 (9)	0.0186 (9)	−0.0015 (10)
O2	0.1073 (12)	0.0620 (9)	0.0817 (11)	−0.0251 (8)	0.0283 (10)	−0.0176 (8)
O3	0.0430 (6)	0.0450 (7)	0.0615 (8)	−0.0013 (5)	0.0057 (6)	0.0015 (6)
O4	0.0452 (7)	0.0801 (10)	0.0982 (12)	−0.0049 (7)	0.0122 (7)	0.0021 (9)
N2	0.0624 (10)	0.0496 (9)	0.0693 (12)	−0.0064 (8)	0.0080 (9)	0.0057 (8)
N3	0.0700 (11)	0.0718 (11)	0.0558 (11)	0.0022 (9)	0.0071 (9)	−0.0034 (9)
N1	0.0473 (8)	0.0420 (7)	0.0536 (9)	0.0043 (6)	0.0056 (7)	0.0033 (7)
C5	0.0488 (9)	0.0452 (10)	0.0551 (12)	0.0015 (8)	0.0040 (9)	0.0065 (8)
C4	0.0565 (11)	0.0639 (12)	0.0595 (13)	0.0037 (10)	0.0109 (10)	0.0096 (10)
C2	0.0551 (11)	0.0536 (11)	0.0570 (12)	0.0043 (8)	0.0002 (9)	−0.0004 (9)
C11	0.0676 (13)	0.0759 (14)	0.0794 (16)	−0.0104 (11)	−0.0022 (12)	−0.0129 (12)
C6	0.0461 (9)	0.0476 (10)	0.0618 (12)	0.0042 (8)	0.0119 (9)	−0.0012 (9)
C7	0.0482 (9)	0.0444 (9)	0.0544 (11)	−0.0031 (8)	0.0073 (8)	−0.0037 (8)
C8	0.0717 (13)	0.0701 (13)	0.0609 (13)	−0.0068 (10)	0.0190 (11)	−0.0045 (10)
C9	0.0445 (9)	0.0612 (11)	0.0486 (11)	0.0040 (9)	0.0015 (8)	0.0070 (9)
C10	0.0706 (13)	0.0668 (13)	0.0820 (16)	0.0185 (11)	0.0043 (12)	−0.0038 (12)

### Geometric parameters (Å, º)

**Table:**

O1—N2	1.2276 (19)	C11—H11B	0.9600
O2—N2	1.219 (2)	C11—H11C	0.9600
O3—C9	1.3447 (19)	C6—C7	1.512 (3)
O3—C7	1.448 (2)	C6—H6A	0.9700
O4—C9	1.197 (2)	C6—H6B	0.9700
N2—C5	1.414 (2)	C7—C8	1.506 (2)
N3—C2	1.327 (2)	C7—H7A	0.9800
N3—C4	1.351 (3)	C8—H8A	0.9600
N1—C2	1.357 (2)	C8—H8B	0.9600
N1—C5	1.384 (2)	C8—H8C	0.9600
N1—C6	1.467 (2)	C9—C10	1.485 (3)
C5—C4	1.353 (3)	C10—H10A	0.9600
C4—H4A	0.9300	C10—H10B	0.9600
C2—C11	1.484 (3)	C10—H10C	0.9600
C11—H11A	0.9600		
			
C9—O3—C7	117.93 (13)	C7—C6—H6A	109.0
O2—N2—O1	122.85 (18)	N1—C6—H6B	109.0
O2—N2—C5	120.29 (15)	C7—C6—H6B	109.0
O1—N2—C5	116.86 (17)	H6A—C6—H6B	107.8
C2—N3—C4	105.66 (17)	O3—C7—C8	107.95 (14)
C2—N1—C5	104.86 (14)	O3—C7—C6	108.15 (14)
C2—N1—C6	125.45 (14)	C8—C7—C6	110.95 (15)
C5—N1—C6	129.44 (15)	O3—C7—H7A	109.9
C4—C5—N1	107.28 (17)	C8—C7—H7A	109.9
C4—C5—N2	127.87 (17)	C6—C7—H7A	109.9
N1—C5—N2	124.84 (16)	C7—C8—H8A	109.5
N3—C4—C5	110.04 (17)	C7—C8—H8B	109.5
N3—C4—H4A	125.0	H8A—C8—H8B	109.5
C5—C4—H4A	125.0	C7—C8—H8C	109.5
N3—C2—N1	112.15 (17)	H8A—C8—H8C	109.5
N3—C2—C11	123.62 (19)	H8B—C8—H8C	109.5
N1—C2—C11	124.23 (17)	O4—C9—O3	123.20 (17)
C2—C11—H11A	109.5	O4—C9—C10	125.65 (18)
C2—C11—H11B	109.5	O3—C9—C10	111.14 (16)
H11A—C11—H11B	109.5	C9—C10—H10A	109.5
C2—C11—H11C	109.5	C9—C10—H10B	109.5
H11A—C11—H11C	109.5	H10A—C10—H10B	109.5
H11B—C11—H11C	109.5	C9—C10—H10C	109.5
N1—C6—C7	112.86 (13)	H10A—C10—H10C	109.5
N1—C6—H6A	109.0	H10B—C10—H10C	109.5
			
C2—N1—C5—C4	0.4 (2)	C5—N1—C2—N3	−0.6 (2)
C6—N1—C5—C4	174.87 (16)	C6—N1—C2—N3	−175.27 (16)
C2—N1—C5—N2	−178.36 (17)	C5—N1—C2—C11	179.00 (18)
C6—N1—C5—N2	−3.9 (3)	C6—N1—C2—C11	4.3 (3)
O2—N2—C5—C4	179.99 (19)	C2—N1—C6—C7	100.0 (2)
O1—N2—C5—C4	−0.1 (3)	C5—N1—C6—C7	−73.4 (2)
O2—N2—C5—N1	−1.5 (3)	C9—O3—C7—C8	−139.19 (17)
O1—N2—C5—N1	178.41 (17)	C9—O3—C7—C6	100.71 (17)
C2—N3—C4—C5	−0.1 (2)	N1—C6—C7—O3	−55.09 (18)
N1—C5—C4—N3	−0.2 (2)	N1—C6—C7—C8	−173.30 (14)
N2—C5—C4—N3	178.55 (18)	C7—O3—C9—O4	0.4 (3)
C4—N3—C2—N1	0.4 (2)	C7—O3—C9—C10	−178.99 (16)
C4—N3—C2—C11	−179.12 (19)		

### Hydrogen-bond geometry (Å, º)

**Table:**

*D*—H···*A*	*D*—H	H···*A*	*D*···*A*	*D*—H···*A*
C4—H4*A*···O1^i^	0.93	2.45	3.369 (2)	168
C6—H6*A*···O4^ii^	0.97	2.53	3.460 (2)	161

Symmetry codes: (i) −*x*+2, −*y*+1, −*z*; (ii) *x*−1, *y*, *z*.
